# Neuroscience-Informed Creative Group Therapy for Processing Trauma and Developing Resilience During Wartime

**DOI:** 10.3390/jpm16030128

**Published:** 2026-02-25

**Authors:** Sharon Vaisvaser, Yifat Shalem-Zafari, Neta Ram-Vlasov, Liat Shamri-Zeevi

**Affiliations:** 1 School of Society and The Arts, Faculty of Humanities and Social Sciences, Ono Academic College, 1 Academic Avenue, Kiryat Ono 5561201, Israel; yifats@best.ono.ac.il (Y.S.-Z.); netara@best.ono.ac.il (N.R.-V.); liat.shamri@ono.ac.il (L.S.-Z.); 2 Center for Trauma and Resilience, Ono Academic College, 1 Academic Avenue, Kiryat Ono 5561201, Israel; 3 The Interdisciplinary Research Center for Arts and Spirituality: Therapy, Education and Society, Tel Hai College, Upper Galilee 1220800, Israel

**Keywords:** trauma processing, resilience, creative arts therapies, embodiment, self-efficacy, predictive processing, interpersonal neural synchronization, triple network model

## Abstract

Traumatic experiences can disrupt one’s sense of safety, self-efficacy, and relationships. Prolonged stress may lead to anxiety, depression, and diminished agency. The embodied, subjective manifestations of trauma call for personalized therapeutic approaches that address symptoms and foster resilience. Group Creative Arts Therapies (CATs) offer relational aesthetic interventions that promote resilience and trauma recovery. Incorporating body-based methods, movement, materials and visual expression, CATs support interoceptive awareness, multisensory integration, embodiment, and emotional–cognitive processing. This article presents a review and conceptual framework of group CAT interventions during wartime, focusing on challenges related to body awareness, self-efficacy, and autobiographical memory. It examines how creative aesthetic approaches help process trauma and strengthen resilience. Drawing on predictive processing accounts of brain function, the article explores the neuropsychological impact of trauma and how creative group work may modulate related brain mechanisms. Creative techniques can foster bodily anchored self-awareness, self-efficacy and processes of traumatic memory reconsolidation. Aesthetic experiences are associated with changes in brain activation and connectivity through processes of embodiment, externalization, and meaning making. On an intrapersonal level, converging evidence highlights the role of sensory and sensorimotor processing, along with the dynamic interplay between Default Mode, Executive Control, and Salience networks, as conceptualized in the Triple Network Model. On an interpersonal level, the literature points to the dynamics of brain and body synchronization, as emerging phenomena during shared creative engagement. These neurodynamics provide a coherent framework for understanding how creative arts-based psychotherapeutic group work can support trauma processing and the cultivation of resilience.

## 1. Introduction: Trauma and War-Related Ongoing Traumatic Stress

Potentially traumatic events (PTEs) can have a profound impact on mental health and well-being. They arise from exposure to actual death, serious injury, sexual violence, or a threat to one’s or others’ physical integrity by experiencing, witnessing or learning about an event, and repeated or extreme exposure to aversive details of traumatic events (DSM-5). Prolonged traumatic circumstances, such as the 7 October attack and the ongoing war in Israel, have placed a large number of individuals at risk of developing Post-Traumatic Stress Disorder (PTSD), depression, and generalized anxiety disorder (GAD) [1]. Symptomology can include intrusive re-experiencing and intrusive thoughts, such as unwanted memories, distressing images, flashbacks, nightmares, and sensory reliving of the event. Individuals may also experience rumination, hypervigilance, and exaggerated threat monitoring, alongside experiential avoidance of trauma-related thoughts, emotions, bodily sensations, or external reminders. Additional features may involve alterations in cognitions and mood, including persistent negative beliefs about the self or the world, diminished interest or emotional numbing, and excessive negative emotions such as shame, blame, guilt, and fear [2]. These symptoms often co-occur with dysregulation of arousal and bodily states, contributing to impairments in functioning, relationships, and sense of agency. These can also be induced by media coverage and social media platforms [3].

Continuous Traumatic Stress (CTS) reflects ongoing exposure to wartime threats and repeated stressful events in daily life that can lead to a broad spectrum of emotional and behavioral reactions [4]. Continual threat to personal safety may explain the considerably high levels of PTSD, depression, and anxiety [1]. Accordingly, wartime conditions are often characterized by heterogeneous and transdiagnostic patterns of psychological distress accompanied by functional impairment, shaped by both trauma exposure and ongoing daily stressors [5]. Clinical evidence further describes the onset of an acute depressive episode following war trauma exposure, characterized by insomnia, abulia, anhedonia, depressed mood, reduced energy, psychomotor disturbances, and marked work and social impairment [6]. Protracted exposure, especially mass casualties and mass traumatization, can disrupt and undermine resilience, underscoring the need for psychological aid to numerous individuals in group interventions that can cultivate resilience.

The present manuscript adopts a narrative and integrative review approach. The literature was synthesized based on conceptual relevance to three intersecting domains: neuroscientific models of trauma and resilience, clinical and psychotherapeutic research on trauma interventions, and empirical and theoretical work in CATs and aesthetic experience. Priority was given to peer-reviewed empirical studies, meta-analyses, and theoretically influential contributions, with emphasis on recent developments alongside foundational models where conceptually necessary. The synthesis was guided by a translational aim to articulate converging mechanisms across brain, body, and relational levels of analysis that may account for the therapeutic impact of group-based CATs in wartime contexts.

## 2. Resilience in the Face of Prolonged Traumatic Events

Broadly defined, resilience is the successful adaptation after trauma, adversity, or stressful life events and taps the dynamics of different peritraumatic factors that comprise and shape the process of coping with the aftermath of traumatic exposure [7].

Resilience has been consistently linked to the capability for effective emotional processing and regulation, reward responsiveness, cognitive flexibility and control, facing fears and active coping, and the ability to harness social support [8]. These psychological factors can contribute to more successful treatment or shield against the development of debilitating post-traumatic stress syndromes. Social support is exceptionally important to maintaining good physical and psychological health when individuals face environmental risks [9]. A study of resilience in Israeli society underlined the importance of community strength and hope [10]. Moreover, a flexible mindset is vital for resilience, because it provides motivation for flexible responding [11]. Flexibility is fostered through creativity, which facilitates social engagement and interaction by encouraging flexible thinking, problem-solving, and the exploration of alternative perspectives [12].

The Creative Arts Therapies (CATs), covering art therapy, dance/movement therapy, drama therapy, psychodrama, music therapy, and poetry/bibliotherapy, use experience-oriented techniques to enable work on fundamental aspects of the self and self-other interactions by tapping the human potential for neuroplasticity [13]. The use of art can actively promote social support and bolster potential resilience resources.

Creative psychotherapy groups utilizing visual art [14,15] and dance/movement techniques [16] as well as combined multimodal approaches [17] were shown to enhance the management of symptoms of psychological trauma, anxiety and depression. The active elicitation of embodied physiological reactivity in artistic engagement may predict greater anxiety reduction promoting emotion regulation [18].

When struggling with trauma or emotional distress, artmaking or engaging in aesthetic experiences can engender transformations [19]. The aesthetic encounter provides a safe and expressive outlet for exploring and confronting complex emotions, and valuable insights into individuals’ emotional landscape [20]. In CATs, the creative process and the resulting artwork promote therapeutic factors of externalization–concretization, embodiment, and symbolization that facilitate affective and cognitive processing, engaging distributed neural systems that integrate multisensory perception, memory, emotion, and meaning-making, reviewed by [21].

Externalization in CATs enables psychological distancing, by cognitively framing the artistic representation. These processes enable the experiencing and embracing of intense emotions tapping into broader and richer emotional repertoires involving negative affect [22], which is crucial in the context of trauma. The therapeutic opportunities for embodied actuality, meaning the direct, lived, here-and-now bodily experience of emotions, sensations, and actions as they unfold in the therapeutic encounter, can induce awareness, and change individuals’ perceptions, thoughts and feelings about the self and others [23]. The use of symbolic and metaphoric expressions in CATs supports a transformative (verbal and non-verbal) narration of experiences that are otherwise difficult to access [24]. The artmaking process, consisting of embodiment, creation, observation, reflection, and meaning-making processes, aims toward a better integration of body and mind [25]. These therapeutic processes are explored in greater detail in the following sections.

## 3. Body- and Movement-Oriented Work to Cultivate Self-Awareness and Process Trauma

CATs use embodied processes to support healing by focusing on bodily sensations and action, sensorimotor interaction with materials, rhythm and sound perception, prosodic entrainment, embodied imagery, and autonomic affective regulation. Body movement reflects and influences emotional states and plays a vital role in integrating physical, emotional, mental, and social functions [26]. All CATs engage the body through meaningful actions that help re-establish sensorimotor feedback loops to foster agency, self-awareness, and self-environment relationships [16,27]. The experiential and sensory nature of CATs make them effective in addressing wordless and sensory trauma memories [24], enabling individuals regain control, self-esteem, and empowerment [28].

Awareness of one’s own states is an important part of cognition and emotion regulation. Emotional Awareness is defined as the ability to recognize, understand and manage emotions [29]. Emotions are body-based and relate to the sense of the physiological state of the entire body [30]. Body awareness occurs simultaneously with emotional processes. It relies on interconnected processes of interoception, i.e., perceiving internal sensations by imbuing these stimuli with emotional weight and using them to guide attention and behavior; proprioception, i.e., awareness of body position in space and an interior sense of the relational positioning of body parts; and exteroception, i.e., receiving and transmitting external information.

The relationship between body and emotional awareness is crucial to the development and maintenance of a sense of self as an integrative construct that organizes sensorimotor, affective, cognitive, and social processes. Strong evidence links interoception to emotional experience through shared neural substrates involved in body regulation and emotion [31]. Disruptions in body and emotional awareness are linked to depression, anxiety, and PTSD [32,33].

The roles of body awareness and actualization, referring to the process by which bodily sensations, emotions, and intentions become experientially expressed through movement and action, underscore the importance of embodied work both as a diagnostic aid and as a component of therapeutic strategies [34]. In trauma therapy, incorporating movement can stimulate brain plasticity and aid regulation, through movement reflection and kinesthetic empathy [35,36]. Movement reflection refers to the therapeutic practice of observing and mirroring movement patterns in order to enhance bodily awareness and emotional insight. By externalizing and reflecting the movement qualities (e.g., tension, contraction, expansion, rhythm), individuals can recognize implicit affective states and experiment with alternative embodied responses. Kinesthetic empathy describes the embodied resonance that arises when a therapist or group member attunes to and mirrors another’s movement, facilitating nonverbal understanding and co-regulation. In treatment, this process can strengthen relational safety, support affect regulation and foster corrective embodied experiences through shared rhythmic and motor synchronization. Integration can also be achieved through movement metaphors for emotional and psychological processing at the symbolic level [26].

## 4. Trauma and Creative Self-Efficacy

Creativity, the ability to reorganize knowledge, information, cues, facts and skills in a person’s reservoir to generate new ideas or useful solutions [37], enters into virtually all aspects of human life. Creativity has been linked to subjective, emotional, psychological and social aspects of well-being and is the basis of interventions supporting well-being [38]. Creative self-efficacy, defined as individuals’ belief in their ability to produce creative outcomes, has been linked to the positive relationship between trauma exposure and posttraumatic growth [39]. Within this framework, emotional creativity denotes the capacity to experience and express emotions in novel and adaptive ways, whereas cognitive creativity involves flexible thinking, cognitive reappraisal, and the generation of new perspectives in response to adversity. Both dimensions have been associated with posttraumatic growth and psychological adjustment following trauma exposure.

Indeed, self-efficacy, fundamental to agency, influences how individuals respond to PTEs [40]. Perceived self-efficacy encapsulates an individual’s self-belief in their ability to exert control over a broad spectrum of challenging or novel tasks and navigate through adverse events. In the context of trauma, this self-belief refers to the perceived capability to manage one’s personal functioning and the myriad environmental demands occasioned by the traumatic event. Low levels of self-efficacy have been found across various trauma-exposed populations, leading to a heightened risk of developing PTSD or other mental health issues, whereby its experiential increase may play a key role in the recovery process [41]. Positive changes brought about by posttraumatic growth, i.e., remodeling values and life goals, expanding social support, enhancing self-awareness, and improving self-efficacy, can catalyze resilience [42]. Accordingly, self-efficacy is considered a resilience resource or protective factor, capable of mitigating the impact of adversity and aiding in the recovery from traumatic experiences during wartime [43]. Higher perceived self-efficacy has been associated with reduced posttraumatic distress, more adaptive coping strategies, and greater psychological adjustment in populations exposed to war and terror. Social support represents another central resilience resource. Perceived availability of emotional, instrumental, and communal support has been shown to buffer stress responses, reduce symptom severity, and enhance adaptive functioning in contexts of armed conflict and forced migration. In the context of war or terror-related trauma, which profoundly disrupts individuals’ sense of control, competence, and social connectedness, interventions aimed at strengthening self-efficacy and reinforcing supportive social networks may play a critical role in promoting psychological recovery and sustained resilience [43].

Creative self-efficacy may therefore function at the intersection of individual agency and social connectedness. When individuals perceive themselves as capable of engaging their emotional and cognitive creativity, they may reconstruct meaning, expand relational possibilities, and interact more adaptively with their social environment, thereby strengthening both personal resilience and interpersonal resources. Increased creativity may constitute a manifestation of posttraumatic growth, with the mediating role of interpersonal relationships and perceptions of new possibilities [44].

Creative activities have been associated with psychological and social well-being. Among trauma survivors, engagement in creative activities may facilitate the processing of traumatic memories while simultaneously boosting self-esteem, self-confidence, and creative self-efficacy, and has been linked to reductions in anxiety, depression, and stress [14].

## 5. Aesthetic Experiences as Facilitators of Predictive Processing

Building on the role of creativity and self-efficacy in trauma recovery, predictive processing (PP) offers a neurocomputational framework for understanding how aesthetic engagement may modulate maladaptive predictions. The PP framework of brain function posits that the brain continually generates predictions about the self and the world and then updates and refines them to minimize the mismatch between expected and received sensory inputs. These processing mechanisms that involve perceptual, emotional and cognitive inferential processes enable individuals to adjust to dynamic, complex environments, by formulating expectations (mostly unconsciously) that guide the processing of sensory information [45].

The PP framework has been applied to trauma, which is considered to cause malfunctions in the brain’s generative predictive models [46,47]. Traumatic circumstances can induce maladaptive predictions, such as conceptual representations that include the belief that the world is inherently dangerous, which can constitute aberrant hyperpriors, namely overly rigid beliefs that dominate perception and resist updating despite contradictory evidence. These reinforce avoidant behavior and can contribute to the maintenance of intrusions [48]. Traumatic experiences may bias perceptual and interoceptive bottom-up sensory priors, expectations about incoming sensory signals based on past experience, thus triggering intrusive reexperiencing [49]. Aesthetic experiences can facilitate the predictive brain. Through creative expression and impression, these experiences are laden with tension, an interplay between predictability and uncertainty. This tension generates salient yet tolerable prediction errors, inviting exploratory active inference as individuals search for hidden meanings in the unfolding experience, thereby modulating the dynamics supporting predictive processing [21]. In this way, prior expectations can be flexibly updated.

Empirical research in trauma neuroscience and neuroaesthetics supports the relevance of predictive processing mechanisms to both traumatic memory and aesthetic experience. Direct experimental studies examining these mechanisms specifically within group-based CATs remain limited. The present integration therefore synthesizes convergent findings across adjacent domains to propose a mechanistically informed framework.

The resilience mechanisms that foster remission and mitigate the adverse effects of stress and trauma may be affected by the extent of plasticity of memory control circuits that are dependent on PP [50]. The use of creative therapeutic techniques may update internally generated predictions that draw on memory traces in the process of reconsolidation. During reconsolidation, previously encoded memories become labile and susceptible to modification upon reactivation. This process may also occur within the implicit realm, that is, at nonconscious affect-laden levels of memory that are not yet verbally articulated [23]. During emotional reactivation, when assimilated with new emotional experiences, memory mismatches or prediction errors allow the situation to be experienced and understood in a different way. By adding safe elements to the memory, working through the emotional consequences of the new learning in a variety of contexts, creative therapeutic processes can enable a more coherent narrative, i.e., embodied predictive models of the body in the world [23].

## 6. Reconstructing Embodied Narratives Through Creative Techniques

Traumatic memories have a profound impact on individuals’ well-being and may be a transdiagnostic feature of multiple mental health difficulties, including PTSD [51]. These can generate a vicious cycle of intrusive and disturbing emotions and cognitions associated with poor elaboration and contextualization [52,53]. Traumatic memories differ from autobiographical memories in their mode of encoding and retrieval. Rather than being integrated into a temporally organized and verbally elaborated narrative, traumatic memories are stored in a fragmented, sensory-bound, and affectively intense manner, with reduced contextual integration [52]. Such disruptions in temporal sequencing, meaning attribution, and self-referential processing may contribute to narrative incoherence, whereby the traumatic event remains insufficiently integrated into the broader autobiographical memory system. As aforementioned, the reactivation of memories can make the memory transiently labile, thus enable a process of memory reconsolidation, where new learning modifies the expression of motor, episodic and emotional aspects of memories [53,54].

Art-based therapies are likely to be advantageous for reconsolidation, both implicitly and explicitly [55]. The CATs provide creative access to nonverbal autobiographical memories while mitigating habitual reactions in a secure environment [55]. These may lead to affective change and a reduction in the emotional repercussion of these memories [56] by providing “opportunities to modulate, sooth, enhance, rewrite, explore, feel, forget or merely reflect upon aspects of the narrative self” [57].

Self-narratives are anchored in embodied lived experiences. In traumatic circumstances, CATs can enable the creation of narratives generated by the body and the art materials. These may be pre-symbolic, implicit, and relational unconscious or unrepresented forms of experience that precede verbal language and explicit representation and have never been narrated [58].

## 7. Creative Work Engages Functional Brain Network Dynamics

Neuroimaging research has identified PTSD-related structural and functional alterations in brain regions involved in emotion processing and memory, including heightened amygdala reactivity, altered prefrontal-limbic connectivity, and disrupted hippocampal-dependent contextual processing [59]. Manifested in the body, trauma has also been linked to alterations in sensory processing, including hyperresponsivity to external stimuli, disrupted sensory gating, impaired multisensory integration, and disturbances in interoceptive awareness, often accompanied by functional alterations in the sensory cortices [60]. Beyond region-specific findings, alterations in resting-state functional brain networks can help account for the complex neurobiological mechanisms underlying psychiatric disorders including PTSD [61,62]. These alterations in large-scale networks may disrupt predictive mechanisms, playing a role in the etiology of trauma symptoms. The Triple Network Model defines three main networks that play a particularly important role in mental health: the Default Mode Network (DMN), the Executive (frontoparietal) Control Network (ECN) and the Salience Network [61].

The DMN, anchored in the medial prefrontal cortex and posterior cingulate cortex, and includes the inferior parietal lobule and hippocampus, is important for self-referential mental activity, the regulation of emotional states, mind-wandering and autobiographical recollection of previous experiences [63]. The ECN, anchored in the dorsolateral prefrontal cortex and posterior parietal cortex, plays a role in working memory, executive functioning, cognitive control, error encoding, explicit attention, and decision-making in goal-directed behavior [61]. The salience network, anchored in the insula, anterior cingulate cortex and amygdala, with prominent subcortical nodes in the affect and reward processing regions such as the amygdala and ventral striatum, is important for stimulus-driven attention allocation, interoceptive inference and awareness, as well as dynamic switching between the DMN and ECN, enabling transitions from self-referential mental processes to current task goals [61,64].

The inability to switch effectively between these neurocircuits in response to environmental demands may be one of the fundamental mechanisms leading to vulnerability to mental health symptoms, such as the emergence of PTSD symptoms [65], as well as in the depressive spectrum [66]. Dysfunction of the harmonious functioning of these core networks in states of anxiety and depression was suggested to be reversible [67].

Creativity and aesthetic experiences have been associated with dialectical interactions between the DMN and ECN, as well as increased activity and connectivity between the DMN and both the sensorimotor cortices and the salience and reward networks, reviewed by [21]. These alterations in neural circuits were related to the therapeutic effects including emotional processing and regulation [68]. Neuroimaging studies of increased self-efficacy also implicate the crosstalk between regions of the medial and lateral prefrontal cortex [41]. Studies have pointed to possible correspondences between neural and psychological outcomes of CATs interventions, with preliminary evidence suggesting modifications in functional connectivity between the major nodes of the default, executive and salience networks in PTSD [69]. To reestablish brain dynamics, the interlaced processes of active art engagement and concretization, emotional processing, perspective taking and reframing within the context of a therapeutic relationship may plausibly contribute to symptom improvement and more adaptive brain functioning [70].

## 8. Creative Relational Synchronization

Psychotherapeutic work in a recurring group setting may facilitate Interpersonal Neural Synchronization (INS), i.e., the coupling of brain activity between people interacting with one another [71]. Interpersonal synchronization involves the alignment of physiological, behavioral, emotional, and brain states during social interactions. Reduced synchrony across brains is a core feature of psychopathology [72] and increased INS has been associated with the facilitation of social interaction and communication and the development of resilience [73]. Spontaneous neural synchrony between individuals was shown to arise during collaborative drawing [74] and in shared movement [75,76,77]. CATs inherently involve behavioral entrainment, or rhythmic synchronization, which can strengthen reciprocity and mutual engagement, supporting integration, relational processes and communication. Creative work may facilitate neural synchrony driven by shared attention to external stimuli (i.e., artwork, sound or musical cues, a prop) or directly mediated by the bodily-anchored person-to-person communicative signals. Communicative aesthetic group “moments of meeting” may induce enhanced INS and possibilities for shared narratives and mutual predictability [21,78]. Social brain perspectives emphasize that language operates within broader neural systems supporting social cognition and interpersonal understanding, integrating both verbal and non-verbal cues in dynamic interaction [79]. In group therapy contexts, this integration of embodied expression and dialogue may facilitate interpersonal attunement and shared meaning-making.

## 9. Creative Group Therapy for Processing Trauma and Developing Resilience

In group therapy, individuals can share experiences, build connections, and experience emotional growth, while the group setting itself facilitates the processing of distressing experiences, reduces isolation, and enhances resilience. Group therapy integrating art and movement-based interventions provides expressive and somatic pathways to access and reorganize traumatic material [24]. Movement helps participants reconnect with their bodies, while the incorporation of visual art enables the externalization of emotional states, insights and group cohesion.

### 9.1. Outcome Literature

Meta-analytic findings indicate statistically significant improvements in trauma-related symptoms, depression, and anxiety following arts-based interventions, with effect sizes generally in the small-to-moderate range for common mental health outcomes and larger for PTSD-specific symptoms, often relative to waitlist, usual activities, or treatment-as-usual control conditions. However, substantial methodological heterogeneity, frequent reliance on small samples, variation in control group types, and the limited number of high-quality randomized controlled trials constrain the strength and generalizability of these conclusions [80]. Group-based visual arts therapy has been linked to declines in depression, anxiety and stress, alongside increases in positive outcomes (e.g., quality of life), potentially driven by nonspecific relational aesthetic effects, namely the shared emotional, sensory, and meaning-making processes that emerge through engagement in joint aesthetic experiences within the group [15]. Multimodal group interventions integrating arts-based practices with facilitated discussion and reflective processing have been associated with enhanced emotional expression and regulation, trauma processing, identity reconstruction, and well-being [17,81]. This combined approach may support emotion awareness and regulation and promote neuroplasticity in brain circuits involved in emotional memory and self-perception.

The incorporation of mind–body exercises (e.g., yoga) as adjuvant treatments, has been shown to significantly improve PTSD symptoms, depression, and anxiety [82]. Research further suggests that mindfulness training in natural environments may be associated with positive psychological outcomes, particularly in less formal, everyday practice contexts that involve embodied engagement [83]. Moreover, studies on Mindfulness-Based Art Therapy (MBAT) demonstrate the potential complementarity between attentional regulation of present-moment experience and creative artmaking processes, suggesting neurobiologically grounded mechanisms relevant to the treatment of anxiety and related conditions [84].

In contrast to mindfulness and other mind–body practices, Creative Arts Therapies emphasize active symbolic transformation, relational co-regulation, and embodied externalization. These processes may modulate maladaptive predictions through generative aesthetic engagement, potentially facilitating trauma-related memory updating beyond attentional modulation and emotion regulation. Direct comparative trials remain limited, and further research is needed to delineate differential mechanisms.

### 9.2. Personalized Treatment Approaches

Creative approaches to processing trauma and developing resilience should consider individual differences in susceptibility and responsiveness to environmental stimuli. Certain inborn biological and developmental factors make some individuals more or less sensitive to environmental influences, shaping how they are affected by both adverse, vulnerability-enhancing conditions and supportive, resilience-promoting environments [85]. In this context, environmental sensitivity was suggested to be reflected in heightened salience network engagement, stronger functional coupling between the salience network and both the DMN and the ECN. Such network dynamics may facilitate automatic orienting to both aversive and rewarding stimuli, alongside greater flexibility in shifting attentional states [85].

CATs are personalized and process-oriented, attending to individual differences in response to aesthetic experiences, shaped by internal factors, personality traits, proneness to aesthetic reactions, expertise, emotional capacities, relational dynamics, and contextual factors, such as socio-demographic factors, educational, or cultural background [19,21]. Variations in body and emotional awareness, emotional differentiation, creativity, and cognitive-behavioral flexibility influence how individuals engage with and derive meaning from artistic processes. CATs explicitly address and explore these differences across expressive modalities, with emerging empirical methods for assessing aesthetic experience in therapy [21].

Trauma presentations in wartime contexts vary substantially in severity and symptom configuration. Careful clinical assessment is therefore essential to determine the appropriateness of group-based interventions. For individuals presenting with high levels of dysregulation or acute instability, arts-based group therapy should be carefully considered and integrated with additional therapeutic modalities and individualized support. Such differentiation underscores the importance of tailoring interventions to clinical needs rather than assuming uniform suitability across trauma profiles.

### 9.3. Limitations and Future Directions

The implementation of neuroscience-informed creative group therapies in wartime settings requires attention to feasibility, cultural adaptation, and ethical safeguards. Resource limitations, ongoing threat, and high levels of distress necessitate structured yet flexible formats that can be delivered in community-based settings. Therapists require training in trauma-informed care, group facilitation, and affect regulation under high-stress conditions. Cultural sensitivity in the use of symbolic and embodied expression is essential, as cultural context shapes how traumatic experiences are interpreted, narrated, and emotionally communicated within group settings. While group-based creative interventions may offer scalable responses to widespread distress, further implementation and outcome research is needed to establish optimal delivery models.

Future research should advance evidence-based neuroscience on individually tailored creative group therapies by examining brain circuitry and brain–body–mind interactions. Mobile Brain-Body Imaging (MoBI), especially in hyperscanning settings, i.e., experimental paradigms in which neural activity from two or more individuals is recorded simultaneously during real-time interaction, offers cutting-edge insights into the dynamic interplay between the brain, body, and social environment [86]. Combining MoBI neural biomarkers with patients’ subjective experiences in mixed-method designs may offer a more holistic understanding of psychotherapeutic outcomes [87].

Notably, PTSD is a severe and often long-lasting condition, and many individuals experience persistent or recurrent symptoms, particularly under ongoing stress. Accordingly, interventions in wartime contexts should be understood as supporting symptom modulation and resilience rather than implying definitive cure.

## 10. Summary and Conclusions

This manuscript presented a conceptual framework for group therapy in wartime to process traumatic experiences and cultivate resilience, through personalized approaches, using creative techniques. The therapeutic factors of CATs prompt processes of embodied actuality, elicited through lived experiences that foster body and emotional awareness, facilitate externalization for psychological distancing and acceptance of intense emotions, and engage symbolic and metaphoric modes of expression. The creative process can promote predictions of self-efficacy, and self-narratives that are reconsolidated and reconfigured through expressive techniques.

The embodied, active, generative, predictive ways the brain operates, ground the use of creative artistic approaches in therapeutic work. The incorporation of expressive, bodily anchored forms of communication with the self and others, integrating both verbal and non-verbal modes of processing autobiographical information, can activate and enhance connectivity across large-scale brain networks fundamental to self-integration. The integration of movement and creative techniques can induce interpersonal synchrony on neural, behavioral, and psychological levels, supporting the neurodynamic processes involved in coping with trauma and fostering greater resilience.

The present framework presents integrative and hypothesis-generating insights into group-based CATs in wartime contexts. Direct longitudinal and neurobiological investigations through multimodal and implementation research are still needed. By articulating convergent mechanisms across brain, body, and relational processes, the therapeutic model provides a structured translational perspective for advancing evidence-informed creative group interventions in contexts of sustained threat.

## Data Availability

No new data were created or analyzed in this study. Data sharing is not applicable to this article.
